# Assessing the efficiency of the bovine brucellosis surveillance-control system in a disease-free context through agent-based modelling

**DOI:** 10.1186/s13567-025-01549-1

**Published:** 2025-06-17

**Authors:** Sofia Mlala, Sébastien Picault, Carole Sala, Pierre Villard, Jean-Luc Vinard, Viviane Hénaux

**Affiliations:** 1 University of Lyon, French Agency for Food, Environmental and Occupational Health & Safety (ANSES), Laboratory of Lyon, 31 Avenue Tony Garnier, 69364 Lyon Cedex 07, France; 2 INRAE, Oniris, BIOEPAR, 44300 Nantes, France

**Keywords:** Cattle, abortion, surveillance, brucellosis, modelling, epidemiology

## Abstract

In France, bovine brucellosis is subject to stringent surveillance to ensure early detection of re-emergence and maintain the country's disease-free status. However, the cost-efficiency of such surveillance systems has rarely been assessed. This study aimed to: (1) evaluate the detection delay of bovine brucellosis for current and alternative surveillance systems following its reintroduction; and (2) estimate the associated surveillance and control costs. A mechanistic, stochastic, agent-based simulation model was developed to represent the spread of bovine brucellosis within and between cattle farms in mainland France after the introduction of an infected animal. The results showed: (1) improved efficiency (considering both effectiveness and cost) when suspending testing at introduction; (2) reduced efficiency when annually screening all adults in one-third of suckler herds compared to 20% of adults in all suckler herds; and (3) slightly improved efficiency when reporting abortion series instead of all individual cases. The current surveillance system detected infection within a median delay of 49–51 weeks, with a median of one infected farm at confirmation, regardless of the epidemiological context. For alternative systems, detection occurred within a median of 40–99 weeks, with one to three infected farms. These findings suggest that, under model assumptions, all surveillance systems maintained a relatively low number of infected herds. Our study provides cost-effectiveness information that will help stakeholders compare the efficiency of alternative scenarios, improve the current surveillance system and make relevant decisions about the allocations of resources.

**Supplementary Information:**

The online version contains supplementary material available at 10.1186/s13567-025-01549-1.

## Introduction

In France, surveillance and control systems for regulated bovine diseases (e.g., tuberculosis, enzootic leukosis, brucellosis) were established in the 1960s–1970s, when their health and economic impacts were significant. Despite achieving “officially free” status and improved epidemiological conditions, these systems have undergone little modification. Maintaining such systems remains costly, particularly for diseases absent from French territory with low—though non-zero—reintroduction risks [1]. In a context of constrained resources for veterinary public health, assessing and potentially improving the efficiency (considering both effectiveness and cost) of these systems is essential.

This study examines the surveillance and control system for bovine brucellosis in France, where the disease is considered exotic. Bovine brucellosis is a contagious disease caused by *Brucella abortus*, *B. melitensis*, or *B. suis*. In France, the disease is strictly regulated due to its significant economic impact on the cattle industry and its threat to human and animal healths. In domestic ruminants, infection typically causes abortion outbreaks but can also lead to sporadic abortions [2, 3]. Less commonly, brucellosis manifests as orchitis, hygromas, or arthritis (inflammation of the testicles, bursae, or joints, respectively) [4]. Currently, bovine brucellosis is eradicated in several countries in northern and western Europe, Oceania, Japan, and Canada but remains endemic in the Middle East, the Mediterranean region, sub-Saharan Africa, Central and South America, and Central and Southeast Asia [5].

A European directive mandates that Member States classified as “bovine brucellosis-free” continuously demonstrate that at least 99.8% of cattle holdings maintain officially free status. This requires a surveillance system capable of detecting at least one infected holding nationally within a year if the proportion of infected holdings reaches or exceeds 0.2% [6]. The system also aims for early detection to minimize the number of infected holdings at the time of detection, thereby reducing transmission risk to humans (via raw milk products or contact with infected materials) and limiting the scale and cost of animal health measures. Infected holdings undergo total culling and livestock renewal upon confirmation of infection. In France, bovine brucellosis surveillance combines: (1) clinical surveillance for early detection through mandatory reporting of all abortions, and (2) programmed surveillance to confirm disease-free status, including annual screening of 20% of adult female cattle (above 24 months) in all suckler herds, annual bulk tank milk testing in all dairy herds, and screening of cattle introduced into herds under certain conditions (e.g., from at-risk herds or following transport exceeding six days) [7]. Across Europe, abortion surveillance and routine herd screening are standard, though technical methods and testing protocols vary by epidemiological context [8]. Movement testing is conducted in half of European countries, with few implementing additional measures, such as routine testing at bull stations and slaughterhouse screening.

Since achieving disease-free status in 2005, France has recorded three outbreaks of bovine brucellosis: two in 2012 and one in 2021. In each instance, only one farm was infected, and disease spread was rapidly contained through sanitation measures. Two of these outbreaks were linked to a wildlife reservoir in an infected ibex population in the Alps, which remains under close monitoring and regulation [9–11]. Despite these limited outbreaks, the cost of bovine brucellosis surveillance remains high relative to the disease-free status. In 2013, the annual cost was estimated at €17 million, with €3.7 million borne by the government and €13.3 million by the farming sector [12]. Clinical surveillance has limited sensitivity, with fewer than one-third of detected abortions being reported [13]. Concurrently, budgetary and human resources allocated to animal health surveillance in France have declined over time. This raises the need to evaluate the performance of the current surveillance-control system and explore resource optimization in light of the epidemiological situation. This study specifically addresses: (1) the detection delay of bovine brucellosis following reintroduction under current and alternative surveillance systems, and (2) the associated costs of surveillance and control. Given the relatively slow inter-farm spread of bovine brucellosis [14] compared to diseases like foot-and-mouth disease [15], we propose a new surveillance paradigm: aiming to detect the disease within a reasonable timeframe rather than at the first case. This approach could lower surveillance costs while maintaining minimal risks to human health and limiting the impact and expense of control measures.

 A few studies have analyzed brucellosis outbreaks in European countries [14, 16, 17]. In France, several efforts have been made to improve the surveillance-control system. The French Platform for Epidemiological Surveillance in Animal Health (ESA Platform) has worked on enhancing the effectiveness and relevance of abortion reporting [18]. Another study evaluated the sensitivity of the current and alternative surveillance systems in maintaining France's officially free status using the scenario tree method [19]. Building on this work, our study aims to assess the efficiency of the surveillance-control system for bovine brucellosis through a modeling approach. Although previous studies have used mathematical models to evaluate disease spread among herds and the effectiveness of surveillance and control measures in various epidemiological contexts [20–23], they did not address cost-effectiveness. To fill this gap, we developed a new epidemic-economic model to simulate (1) farm management practices by farmers; (2) the spread of bovine brucellosis within and between farms; and (3) the detection of infections by the surveillance system.

## Materials and methods

### General presentation of the model

An agent-based model (ABM) was developed to evaluate the efficiency of the bovine brucellosis surveillance and control system in a disease-free context. It simulates disease dynamics within and between farms, integrating farm management practices, infection spread, and surveillance activities. The model is initialized using pre-processed data from national databases to parameterize farm characteristics and demographics. The ABM approach was selected for its capacity to account for the complexity and heterogeneity of cattle farming systems, which are difficult to capture with traditional compartmental models. This framework allowed detailed representation of farm-specific management practices, infection dynamics, and surveillance protocols applied at the individual animal level, including sequential tests or abortion events.

This individual-based, stochastic, discrete-time mechanistic model was built using the EMULSION framework [24], an open-source platform for constructing complex epidemiological models encompassing components such as infection, demography, and surveillance. The EMULSION tool was chosen because (1) it provided an existing, effective framework, avoiding the need for model development from scratch; (2) it facilitated communication through model descriptions that were independent of computer code; and (3) it enabled direct interaction with the framework developer for model design and modification.

The model includes two main entities: cattle farms and individual female bovines (male bovines were excluded for simplicity). The parameters for these entities are listed in Table 1. Each farm is characterized by its typology (e.g., dairy or suckler), initial number of bovines, and age structure, based on data from real French farms. Each female bovine is described by variables such as age, reproductive status, lactation status, and brucellosis infection status.

**Table 1:** **Definitions, names and values of model parameters.**

Entity	Variable	Definition of the parameter	Value	References
Bovine	*proba_successful_insemination*	Weekly probability of insemination	0.95	[51]
Bovine	*dur_gestation*	Gestation period	41 weeks	model assumption
Bovine	*dur_NoGestation*	Minimum time from calving or abortion to insemination	14 weeks	model assumption
Bovine	*proba_abortion_default*	Weekly probability of abortion for a healthy female (throughout gestation)	0.0046	[51]
Bovine	*proba_abortion_default*	Weekly probability of abortion during the first four months of gestation for an infected female	0.0046	[51]
Bovine	*proba_abortion_infected*	Weekly probability of abortion in the last five months of gestation for an infected female	0.0779	[2, 50]
Bovine	*dur_YoungerJuvenile*	Age of first insemination (randomly selected within the value range)	[62; 66 weeks] in dairy [101; 119 weeks] in suckler	Model assumption
Bovine	*proba_female_calf*	Probability of a cow giving birth to a female calf	0.5	Model assumption
Farm	*proba_sell_to_same*	Probability that a herd will sell one or more animals to a herd of the same type (dairy or suckler)	0.7	[46]
Farm	*beta_HighShedder*	Weekly rate of horizontal transmission due to the presence of high shedding females	0.2 or 0.6 depending on epidemiological context	Model assumptions
Bovine	*dur_HighShedder*	Period of high shedding of *Brucella* bacteria by an infected female (after calving or abortion)	9 weeks	Model assumption
Farm	*beta_LowShedder*	Weekly rate of horizontal transmission due to the presence of low shedding females (i.e. infected and outside of their high shedding period)	0.1 * *beta_HighShedder*	Model assumption
Bovine	*proba_vertical_transmission*	Probability of an infectious female contaminating its own calf	0.2	[52]
Bovine	*proba_test_on_purchase_default*	Probability of a purchased animal being tested if the herd of origin does not pose a risk for bovine brucellosis	0.06	Data from the French national cattle register
Bovine	*proba_test_on_purchase_risky*	Probability of a purchased animal being tested if the herd of origin is at risk for bovine brucellosis	1	[7]
Bovine	*Se_RBT*	Sensitivity of the Rose Bengal test (RBT) on a blood sample (probability that an infected animal will test positive)	0.954	[40]
Bovine	*Sp_RBT*	Specificity of the RBT on blood samples (probability that a healthy animal will test negative)	0.977	[40]
Bovine	*Se_ELISA*	Sensitivity of the ELISA (indirect enzyme-linked immunosorbent assay) test on a blood sample	0.960	[40]
Bovine	*Sp_ELISA*	Specificity of the ELISA test on a blood sample	0.938	[40]
Bovine	*Se_CF*	Sensitivity of the complement fixation (CF) test on a blood sample	0.890	[40]
Bovine	*Sp_CF*	Specificity of the CF test on a blood sample	0.835	[40]
Farm	*Se_ELISA_milk*	Sensitivity of the tank milk ELISA test (probability of a herd testing positive in the presence of infected lactating females when the test is performed)	0.979	[40]
Farm	*Sp_ELISA_milk*	Specificity of the tank milk ELISA test (probability that a herd will test negative in the absence of infected lactating females when the test is performed)	0.961	[40]
Farm	*proba_abort_detection*	Probability of an abortion being detected by the farmer	0.3	[13]
Farm	*delay_bacteriological_confirmation*	Time from confirmation of infection to slaughter decision	1 week	Model assumption
Farm	*confirmation_delay_risky*	Time limit for renewal of a serological test, following a positive screening result, in a herd at risk of bovine brucellosis	2 weeks	[34]
Farm	*confirmation_delay_safe*	Time limit for renewal of a serological test following a positive result in a herd not at risk for bovine brucellosis	6 weeks	[34]

As for spatial and temporal resolutions, the model operates on a weekly time step and simulates four production campaigns (July 1 to June 30). It simulates only infected farms, beginning with an index farm where one infected animal is introduced. If trade movements involve the sale of an infected animal, a new farm is added to the simulation.

The key processes of the model, run at each weekly time step, are livestock management, disease transmission, and surveillance. Livestock management includes breeding (insemination, calving, or abortion), lactation, ageing, mortality, and movements to other farms or slaughterhouses. Disease transmission consists in: (1) horizontal transmission within farms, (2) vertical transmission (from infected mothers to offspring in utero) occurring at calving; and (3) between-herd transmission arising from trade movements of infected animals. Surveillance includes an annual serological testing performed on randomly pre-selected dates, abortion reporting, and serological testing of introduced animals.

The model incorporates stochastic elements to reflect variability in farm characteristics, disease dynamics, and surveillance outcomes. For example, herd size and composition are initialized using real-world data, and processes like disease transmission and abortion reporting are modeled probabilistically. Disease dynamics involves interactions within herds (e.g., horizontal and vertical transmissions) and between herds through animal movements.

Key outcomes include detection delay, the number of infected herds, and surveillance costs, which are used to evaluate and compare the performance of different surveillance schemes.

The simulation begins with one susceptible herd, into which a single infected adult bovine is introduced at a random week during the first production season. Variables such as animal age and reproductive status are initialized using data from real French farms. The model is also driven by input data, including farm characteristics, animal movements, and mortality, derived from the national cattle identification database (BDNI). Dates for annual serological testing are sourced from the French veterinary activity database (SIGAL).

The following sections describe the demographic, epidemiological, and surveillance processes incorporated into the model.

### Demographic pattern (farm management)

The simulation model was designed to reproduce herd size dynamics over time as observed in cattle traceability data. The data used for modeling farm management within each simulated herd were obtained in two stages. First**,** the initial number and age structure of cattle in each herd, as well as processes related to mortality, commercial trade, and slaughtering, were modeled using data from reference farms. Second, breeding and lactation processes were modeled based on data extracted from the literature.

For the first stage, to ensure the sample of farms reflected the population covered by the French bovine brucellosis surveillance system, reference farm management profiles were defined using data from the national cattle identification database (BDNI) (details in Additional file 1). A random sample of 1000 farms was selected from those operational between July 1, 2013, and June 30, 2017. Farm types were classified according to the typology developed by Sala et al. [25], which uses thresholds on criteria such as the number of births, slaughtered animals, young animals, and females. Farms were categorized as suckler, dairy, or very small farms. Very small farms were subsequently treated as suckler farms due to their adherence to the same surveillance protocol.

For each selected farm, weekly data over the four-year period were extracted, including the number of female bovines by age class present on the farm, and the number of females (by age class) that died, were slaughtered, sold, or purchased. Farms with incomplete datasets (e.g., missing information on animal numbers or movements) were excluded from the sample.

This approach ensured the model was parameterized with real-world data representative of the surveillance system's target population, enabling accurate reproduction of herd size dynamics over time.

In the second stage, the processes within the model, referred to as “state machines” (Additional file 2), are documented online [26] and graphically represented in Additional file 3. These processes simulate key aspects of cattle life cycles, including reproduction, lactation, and ageing. Within each farm, the reproduction process was modeled with three reproductive states: early gestation (the first four months), late gestation (the last five months), and a non-gestation state. During gestation, the physiological state of each female bovine was updated weekly based on the duration of gestation and the probability of abortion, which varied between healthy and infected animals. For non-pregnant cows, the physiological state was determined by the interval between calving or abortion and the next insemination (or mating), as well as the probability of successful insemination. The lactation process was modeled with two states, lactating and dry. Female bovines transitioned to the dry state two months before calving and returned to lactation following calving. The ageing process categorized animals into three age states: younger juveniles (under 24 months old, before their first insemination), older juveniles (under 24 months old, after their first insemination), and adults (24 months and older). The age of first insemination was randomly assigned to each animal within a range of 101 to 119 weeks. All parameter values for these processes are provided in Table 1. For the purpose of the simulation, a month was assumed to consist of 4.4 weeks.

During the simulations, at each weekly time step, the number and the age of female bovines that died, were sold to other farms, slaughtered, or purchased, were selected using data observed from real herds. Given the critical roles of abortions and vertical transmission (from cow to offspring) in the spread and detection of bovine brucellosis (detailed in the epidemiological and surveillance patterns), births were simulated mechanistically to allow measurement and comparison of the effects of different surveillance strategies. Births could occur throughout the year, with an equal probability for female and male calves.

Sales, loans, and purchases were reproduced on the same dates as in the real data, and animals were selected from the same age category whenever possible. If no animal matched the specified age category from the BDNI data, animals from an adjacent age category were selected. For slaughter, preference was given to non-pregnant females. If none were available, pregnant females from the same age category were chosen. For sales, selection was based solely on age category, with animals being randomly selected within that category regardless of reproductive or lactation status, reflecting real-life scenarios where pregnancy might go undetected. Each farm had a 0.7 probability of selling an animal to a farm of the same production type (Table 1).

Only the sale of infected animals to other farms introduced new herds (the buying farms) into the simulation. Sales of healthy animals were modeled only at the source farm, with the departure of the animals, but did not result in the addition of new farms to the simulation. If a farmer sold more than one infected animal in the same week, these animals were distributed randomly to one or more farms. Buying farms were introduced into the simulation at the time of the movements, selected from farms that recorded a purchase at that time step in the dataset.

Animals purchased were considered infection-free and from herds not at risk of bovine brucellosis, in accordance with French regulations on bovine movements. The age state of each purchased animal was defined based on the data, while breeding and lactating statuses were assigned randomly under constraints to avoid unrealistic situations (e.g., a female being pregnant or lactating before breeding).

Simulations without disease were run to validate the consistency of the simulated population dynamics against the observed dynamics in real farms over the four campaigns of the dataset. The simulated population dynamics closely matched observed data for most farms (Additional file 4). To exclude extreme cases, the 25% of farms with the least consistent results were removed, leaving a final sample of 610 farms. This sample included 40% dairy farms and 60% suckler farms (Additional file 5), reflecting the ratio in the French cattle population (BDNI database).

### Epidemiological pattern

Within-herd transmission occurred through vertical transmission at birth and horizontal transmission following calving or abortion of an infected female. Vertical transmission, where infected cows transmitted the infection to their calves, was modeled with a probability of 0.2 (Table 1). Uterine secretions, placenta, and runt expelled during calving or abortion contain large quantities of bacteria, causing horizontal transmission. The force of infection was assumed to be frequency-dependent, determined by the horizontal transmission rate and the proportion of infected females in the herd. Infected females were classified as high shedders during the two months following calving or abortion and as low shedders at other times, based on existing studies [20, 27] (Table 1). Cattle were assumed to be susceptible to infection at all ages [28], and infected individuals were considered non-recoverable.

To account for variability in horizontal transmission rates associated with strain diversity, the model incorporated a range of plausible values consistent with previous models [20, 21, 29, 30]. Two contrasting values were chosen to represent low and high transmissibility contexts: 0.2 (low transmissibility, resulting in slower infection spread within and between herds) and 0.6 (high transmissibility, leading to faster and broader spread). The horizontal transmission rate for low shedders was set at 10% of the rate for high shedders in both contexts (Table 1).

### Surveillance pattern

Table 2 outlines the possible modalities for the three surveillance measures that make up the current bovine brucellosis surveillance system: screening of reported abortions, annual screening, and screening at purchase. The reference surveillance system, which simulates the current practices [7], incorporates the first modality of each surveillance component: A1 for abortion reporting, P1 for annual screening, and I1 for screening at purchase. Alternative surveillance systems were combinations of different modalities for these three components, as detailed in Table 2. These alternatives were inspired by strategies employed in other European brucellosis-free countries, such as screening every three years (Sweden, Germany), no movement screening (most countries), and reporting series of abortions rather than individual events (Norway, Bulgaria) [8].

**Table 2:** **Modalities of the three surveillance components used to create the different surveillance systems.**

Surveillance components and corresponding abbreviations	Modalities and corresponding abbreviations		
A: Mandatory reporting and screening (antibody and bacteria testing) of abortions	A1: All abortions	A2: Series of two or more abortions over a four-week period, and possibility to report a lone abortion if desired	A3: Same modality as A2, with an awareness campaign to increase the reporting rate
P: Annual screening of adult animals	P1: blood antibody testing of 20% of female adult bovines (24 months and older) in all suckler herds and tank milk antibody testing in all dairy herds	P2: blood antibody testing of all adult bovines in one third of randomly selected suckler herds^a^ and tank milk antibody testing of all dairy herds	–
I: Screening of purchased animals	I1: Blood antibody testing of purchased adult bovines if they come from an at-risk herd or if they were transported for more than six days	I2: No screening of purchased animals	–

^a^This was modelled in our study as a random screening of all suckler herds once every 3-year period.

In the model, the weekly probability of reporting an abortion depended on the total number of abortions detected over the current week and the preceding three weeks (a four-week period). Table 3 details these reporting probabilities for each abortion reporting modality (A1, A2, A3) and production type. The probabilities were categorized based on whether one, two, three, or four or more abortions occurred in the herd during four consecutive weeks (Table 3). The probabilities for reporting the first abortion were based on prior studies [13, 31, 32], which found that dairy farmers had higher reporting rates (~ 41%) compared to suckler farmers (~ 21%). This difference may be explained by technical challenges such as difficulty catching suckler cows at pasture for sampling [13, 31]. In the absence of data, the probabilities for reporting subsequent abortions were set arbitrarily, with an assumed increase as more abortions occurred. Similarly, for alternative reporting modalities (A2 and A3), probabilities for reporting isolated abortions were arbitrarily set low, reflecting the assumption that only a few farmers would continue to report single abortions when reporting series became mandatory.

**Table 3:** **Probabilities of reporting abortion, by reporting modality (A1, A2 or A3).** A1: Mandatory reporting for bovine brucellosis of all detected abortions. A2: Mandatory reporting of series of two or more abortions over a four-week period. A3: Modality A2 with an awareness campaign to increase the reporting rate. The probability of reporting each abortion depends on the number of abortions detected in the herd in four consecutive weeks (one, two, three, and four or more).

Parameter	A1		A2		A3	
	Suckler	Dairy	Suckler	Dairy	Suckler	Dairy
Probability of reporting an abortion, knowing that no other abortion was detected in the herd in four consecutive weeks (including the current week)	0.21^a^	0.41^a^	0.02^b^	0.05^b^	0.02^b^	0.05^b^
Probability of reporting a second abortion in four weeks^b^	0.40	0.60	0.40	0.60	0.50	0.70
Probability of reporting a third abortion in four weeks^b^	0.60	0.80	0.60	0.80	0.70	0.90
Probability of reporting a fourth or more abortions in four weeks^b^	0.80	0.90	0.80	0.90	0.90	0.95

A1: Mandatory reporting for bovine brucellosis of all detected abortions. A2: Mandatory reporting of series of two or more abortions over a four-week period. A3: Modality A2 with an awareness campaign to increase the reporting rate. The probability of reporting each abortion depends on the number of abortions detected in the herd in four consecutive weeks (one, two, three, and four or more).

^a^The probabilities of reporting the first abortion was based on [13, 31, 32].

^b^In absence of observed data: (1) the probabilities of reporting the next abortions were determined arbitrarily, assuming an increasing probability when several abortions occurred; (2) the probabilities of reporting the first abortion in the alternative reporting modalities (A2 and A3) were chosen arbitrarily, assuming a very small number of farmers would continue to report isolated abortions even if only the reporting of series of abortions is compulsory.

For suckler herds, annual blood testing dates were drawn from a normal distribution fitted to observed testing dates in 2013 from the French veterinary activity database (SIGAL). The median testing date was week 10 (early March) with an interquartile range of [5, 18]. As recommended by the Ministry of Agriculture [33], the selection of animals for testing in suckler herds prioritized those at higher risk, i.e., animals introduced since the previous annual screening, which had not beentested at purchase. For dairy herds, testing dates were randomly assigned each year.

Screening at introduction was systematic for cattle from at-risk herds and conducted with a 0.06 probability for cattle from other herds (BDNI). Following French regulations [7], at-risk herds were defined as those with a female turnover rate exceeding 40%, calculated as the ratio of females purchased to the average number of females in the herd over four years. At-risk herds accounted for 6% of the farms in the dataset of 610 farms.

The delay between detecting a positive animal and confirming its sanitary status through additional testing varied by the context of the initial test and the type of herd, as specified by French regulations [34]. For annual screening, confirmation delays were two weeks for at-risk suckler herds, six weeks for other suckler herds, three weeks for at-risk dairy herds, and seven weeks for other dairy herds. For positive results from tests performed at purchase or after reporting abortions (or series of abortions), the confirmation delay was one week.

Detection and confirmation of an infection, under all surveillance systems, resulted in the compulsory culling of all animals in the affected herd, in accordance with regulations [7]. Following culling, the whole simulation was terminated.

### Evaluation of surveillance and control costs

Veterinary fees and unit costs for analyses were derived from [12] (Additional files 6 and 7), with adjustments for inflation in the monetary unit of veterinary medical acts (AMV) [7, 35]. The AMV value in 2020 was used, amounting to €14.18 pre-tax. The cost of surveillance at the national level was calculated by multiplying the annual numbers of interventions and analyses associated with each surveillance system by the unit costs of these interventions and analyses (Additional files 8 and 9).

The annual numbers of interventions and analyses under the current surveillance modalities (A1-I1-P1) were determined using SIGAL data and data from the Interprofessional Milk Analysis Laboratories (LIAL) for 2013, as described in [12]. For modality P2, the number of samples in dairy herds remained the same as in P1. For suckler herds, the number of visits was reduced to one-third of the visits in P1, while the number of samples was adjusted by multiplying the P1 total by 0.33/0.2 to reflect sampling of one-third of herds with 100% of bovines aged 24 months or older being tested (compared to 20% in P1). Each blood sample was assumed to be analyzed using ELISA or Rose Bengal tests, and complement fixation tests in the same proportions as P1.

For modality A2, the number of abortion reports was estimated from the A1 reports, assuming 70% of reported abortions were part of a series (unpublished data) and using a mean probability of reporting a series of abortions of 0.5 (Table 3). For modality A3, the total number of abortion reports in France (Total_reports_FR_A3) was calculated as the average of estimates from surveillance systems using A3. These estimates were derived through cross-multiplication, based on the ratio of simulated abortion reports for A3 (Total_reports_simu_A3) to A2 (Total_reports_simu_A2), multiplied by the estimated total abortion reports in France for A2 (Total_reports_FR_A2):

$$Total\_reports\_FR\_A3= \frac{Total\_reports\_simu\_A3 \times Total\_reports\_FR\_A2}{Total\_reports\_simu\_A2}.$$

For modalities A2 and A3, a single veterinary visit was associated with reporting a series of two or more abortions. Each abortion in the series generated a blood sample and, in the same proportions as A1, samples of placenta, fetus, or genital organ, along with individual ELISA or Rose Bengal tests, complement fixation tests, and bacteriological analyses.

Surveillance costs were categorized by stakeholder: the government covered costs for abortion reporting interventions, while farmers bore the costs for annual screening and testing at introduction. The total cost of each surveillance system was the sum of these stakeholder costs.

Control costs were calculated based on the number and size of infected herds at the time of the confirmation of infection, assuming all infected herds would eventually be detected via epidemiological investigations. Compensation for the slaughter and replacement of cattle in an infected herd was estimated at €1,075 per bovine under 24 months and €2,075 per bovine aged 24 months or more [36, 37]. Disinfection costs for farm buildings were estimated at €15 per animal, based on data from the 2012 outbreaks in France (unpublished data from the Ministry of Agriculture). Total control costs for each simulation were computed by summing the compensation and disinfection costs for all infected herds at the time of confirmation of the infection.

### Simulations

Each simulation began with a single herd randomly selected from the farm sample. An infected adult animal was introduced into this initial herd at a random week during the first production season. Simulations ended as soon as infection was confirmed in a simulated farm, as assessing the control measures post-detection was beyond the scope of this study. It was assumed that all infected farms would eventually be detected either through the surveillance system or epidemiological investigations. Simulations were systematically terminated after four production campaigns if no infection was confirmed.

Seven surveillance systems were tested, each in two epidemiological contexts (low and high transmissibility), resulting in fourteen distinct scenarios. For each scenario, 2000 stochastic replicates were simulated.

The performance of each surveillance system was assessed using two key indicators: (1) the median detection delay, defined as the time between the introduction of infection and the confirmation of infection in a herd; and (2) the median number of infected herds at the time of infection confirmation. The total cost of each scenario was calculated as the sum of the median costs for both surveillance and control measures. Scenario efficiency was evaluated and compared based on differences in the performance indicators and total costs.

### Sensitivity analysis method

We conducted a sensitivity analysis to identify input parameters that significantly influence the variability of the model's main outputs. The following input parameters were tested: horizontal transmission rate, probability of reporting the first abortion, probability of abortion in healthy females, probability of abortion in infected females, probability of selling an animal to a farm of the same production type.

For each parameter, the nominal value, a 20% decreased value, and a 20% increased value were tested. The fractional factorial design method [38] was used to optimize the number of combinations of parameter values, employing the “Planor” package in R [39]. This approach resulted in 81 combinations of values for the five parameters. For each combination, 200 simulations were performed using the current surveillance system. A random sample of 200 cattle farms from the representative farm dataset served as index farms for the simulations, with the same selection used across all parameter combinations.

An analysis of variance (ANOVA) was performed on the model outputs to quantify the contribution of each input parameter to the variability of five key outputs: detection delay; total number of farms infected during the simulation (regardless of production type); number of dairy farms infected during the simulation; number of suckler farms infected during the simulation; number of infected farms at the time of the confirmation of infection (regardless of production type).

The contribution of an input parameter *p* to the variability of an output *o* was calculated using the following formula:

$${C}_{p}^{o}=\frac{{SS}_{p}^{o}+\frac{1}{2}{\sum }_{p{\prime}}{SS}_{p:p{\prime}}^{o}}{{SS}_{tot}^{o}}.$$

Here ${SS}_{tot}^{o}$ represents the total sum of squares for output *o*, ${SS}_{p}^{o}$ the sum of squares related to the principal effect of parameter *p*, and ${SS}_{p:p{\prime}}^{o}$ the sum of squares for the interaction between parameters *p* and *p′*.

## Results

### Detection delay

For all surveillance systems, spontaneous disappearance of infection (i.e., due to infected animals being slaughtered or dying before detection) occurred in 6% of simulations under low transmissibility and 2% under high transmissibility. Conversely, there were simulations where infection persisted throughout the four campaigns and was never detected (Table 4). Such a scenario occurred in 22% of simulations under the current surveillance system in the low transmissibility context and in 21% to 40% under alternative systems. For high transmissibility, these figures were 7% for the current system and 7% to 27% for alternative systems. The number of infected herds at the end of these undetected simulations varied depending on the transmission context, as presented below.

**Table 4:** **Number of infected herds at the time of confirmation of infection. **The table presents, for each surveillance system and each epidemiological context (transmission rate): the number and proportion of simulations where infection was never detected (over 2000 simulations); the median number of infected herds (with the first and ninth deciles in square brackets and the maximum in brackets; the minimum was one infected herd in all designs); the median delay of detection (with the first and ninth deciles in square brackets).

Transmission rate	Output	A1-P1-I1	A1-P1-I2	A1-P2-I2	A2-P1-I2	A2-P2-I2	A3-P1-I2	A3-P2-I2
0.2	Number (proportion) of simulations where infection was never detected	438 (22%)	429 (21%)	532 (27%)	670 (34%)	746 (37%)	680 (34%)	792 (40%)
	Number of infected herds at the time of infection confirmation	1 [1–3] (19)	1 [1–3] (25)	1 [1–7] (33)	1 [1–3] (27)	1 [1–8] (41)	1 [1–3] (18)	1 [1–7] (30)
	Delay of detection	49 [17–147]	50 [18–149]	99 [26–161]	43 [17–111]	88 [26–157]	40 [16–116]	90 [28–155]
0.6	Number (proportion) of simulations where infection was never detected	145 (7%)	157 (8%)	148 (7%)	472 (24%)	536 (27%)	482 (24%)	488 (24%)
	Number of infected herds at the time of infection confirmation	1 [1–8] (57)	1 [1–8] (77)	2 [1–19] (92)	1 [1–11] (71)	3 [1–27] (113)	1 [1–10] (74)	3 [1–27] (96)
	Delay of detection	51 [19–118]	51 [19–118]	76 [27–134]	45 [18–108]	83 [30–141]	45 [18–117]	82 [28–143]

For the current surveillance system, the median detection delay (time from the introduction of infection to its first confirmation in a herd) was 49 weeks under low transmissibility and 51 weeks under high transmissibility. For alternative systems, the median detection delay ranged from 40 to 99 weeks in the low transmissibility context and from 45 to 83 weeks in the high transmissibility context (Table 4). Detection delay showed little variation across different surveillance systems or epidemiological contexts, as indicated by overlapping interquartile ranges. However, several notable observations were made. (1) Stopping tests at introduction (I2) did not impact detection delay (as seen in comparisons between systems A1-P1-I1 and A1-P1-I2). (2) Restricting abortion reporting to series of abortions (A2 and A3) reduced the variability in detection delay between simulations, particularly in the low transmissibility context. (3) Monitoring only one-third of suckler herds annually (P2) tended to increase the detection delay and its variability between simulations, with both trends more pronounced in the low transmissibility context. (4) Variability in detection delay between simulations was lower for all surveillance systems in the high transmissibility context.

### Number of infected herds

In the current surveillance system, when infection was confirmed (ending the simulation), only one herd (the index herd) was infected in at least half of the simulations, regardless of the transmissibility context. In 90% of simulations, up to three herds were infected in a low transmissibility context and up to eight herds in a high transmissibility context. For alternative surveillance systems, in 90% of simulations, up to eight herds were infected in the low transmissibility context, and up to 27 herds in the high transmissibility context (Table 4).

No significant differences in the number of infected herds at infection confirmation were observed across the various surveillance systems. Yet, distinct trends emerged depending on the epidemiological context. In the low transmissibility context, the median number of infected herds at confirmation remained consistent across surveillance systems (one herd—the one with confirmed infection). However, the variability (interquartile range) increased when only one-third of suckler herds were monitored annually (P2). In the high transmissibility context, this same surveillance modality (P2) was associated with increases in both the median number of infected herds and its variability.

Additionally, in the high transmissibility context, combining the P2 and I2 modalities with a shift from reporting all abortions (A1) to reporting series of abortions (A2 and A3) further increased both the median and variability in the number of infected herds. However, increasing the probability of reporting (A3 versus A2) did not affect the number of infected herds.

Across all scenarios, an average of one to six herds were infected before the end of the simulation (whether or not the infection was detected). In the low transmissibility context, this included an average of zero to one infected dairy herd and one to three suckler herds. In the high transmissibility context, the averages increased to one to four dairy herds and two to six suckler herds.

### Detection of infected herds

In the current surveillance system, the median proportion of infected farms, including the index farm, that were detected (i.e., had at least one positive test result) at the time of confirmation of infection was 100%. This was also the case for all alternative surveillance systems, except those involving partial annual screening (P2) in a high transmissibility context. In these cases, the proportion of infected farms detected at the time of confirmation ranged from 41 to 66%.

**Figure 1:**
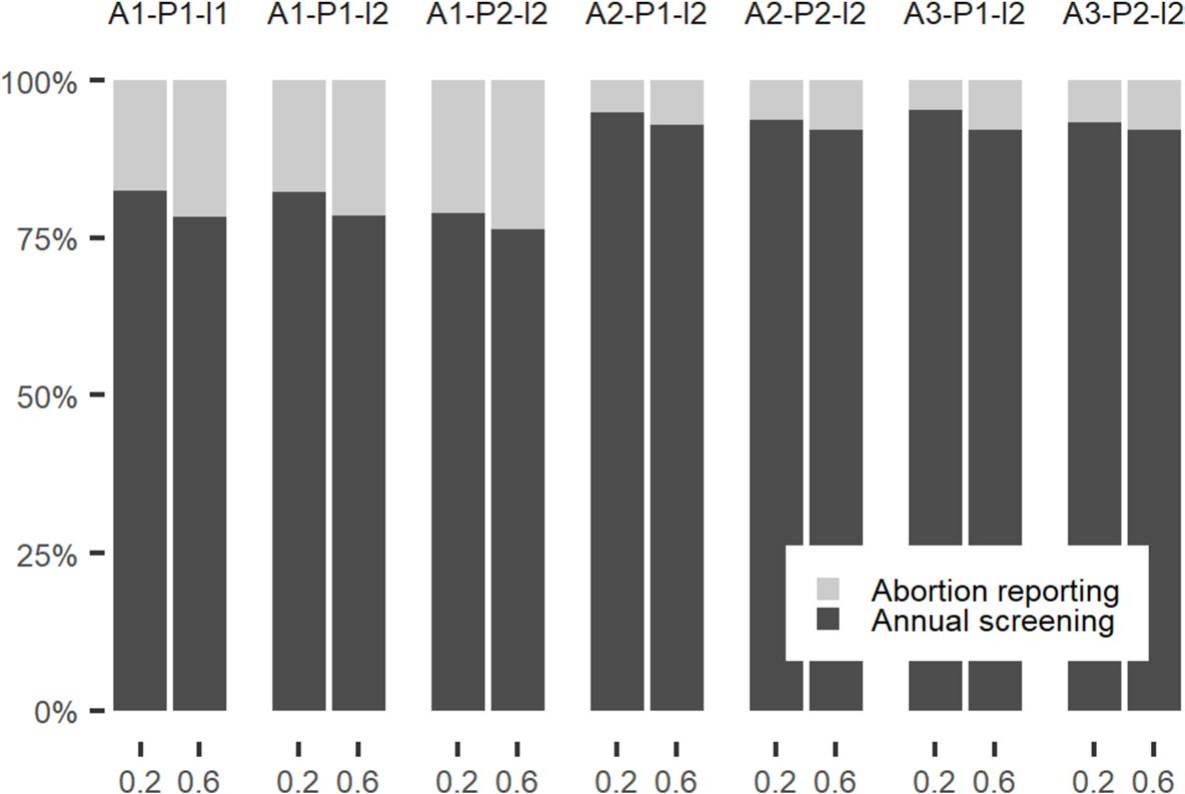
**Proportions of infection detections due to abortion reporting (A1, A2 or A3) and annual screening (P1 or P2), for each surveillance system and epidemiological context (transmission rate).** Control at introduction is not represented on this graph as it almost never enabled detection of the infection (0.07% of detections on average) in the surveillance system where it is present (A1-P1-I1).

Across all scenarios, between 76 and 95% of detected herds were identified through annual herd screening (P1 or P2), while 5–24% were identified via abortion reporting. The proportion of herds detected through abortion reporting decreased by more than 10% when transitioning from reporting all abortions (A1) to reporting series of abortions (A2 and A3). This proportion remained unchanged when the probability of reporting abortion series was increased (A3 versus A2). Virtually no infected herds (0.07% on average) were detected through controls at introduction in the current surveillance system (A1-P1-I1), which was the only system including this modality (I1) (Figure 1). No significant differences in the proportions of infected herds detected were observed between the two epidemiological contexts for any surveillance system.

**Figure 2:**
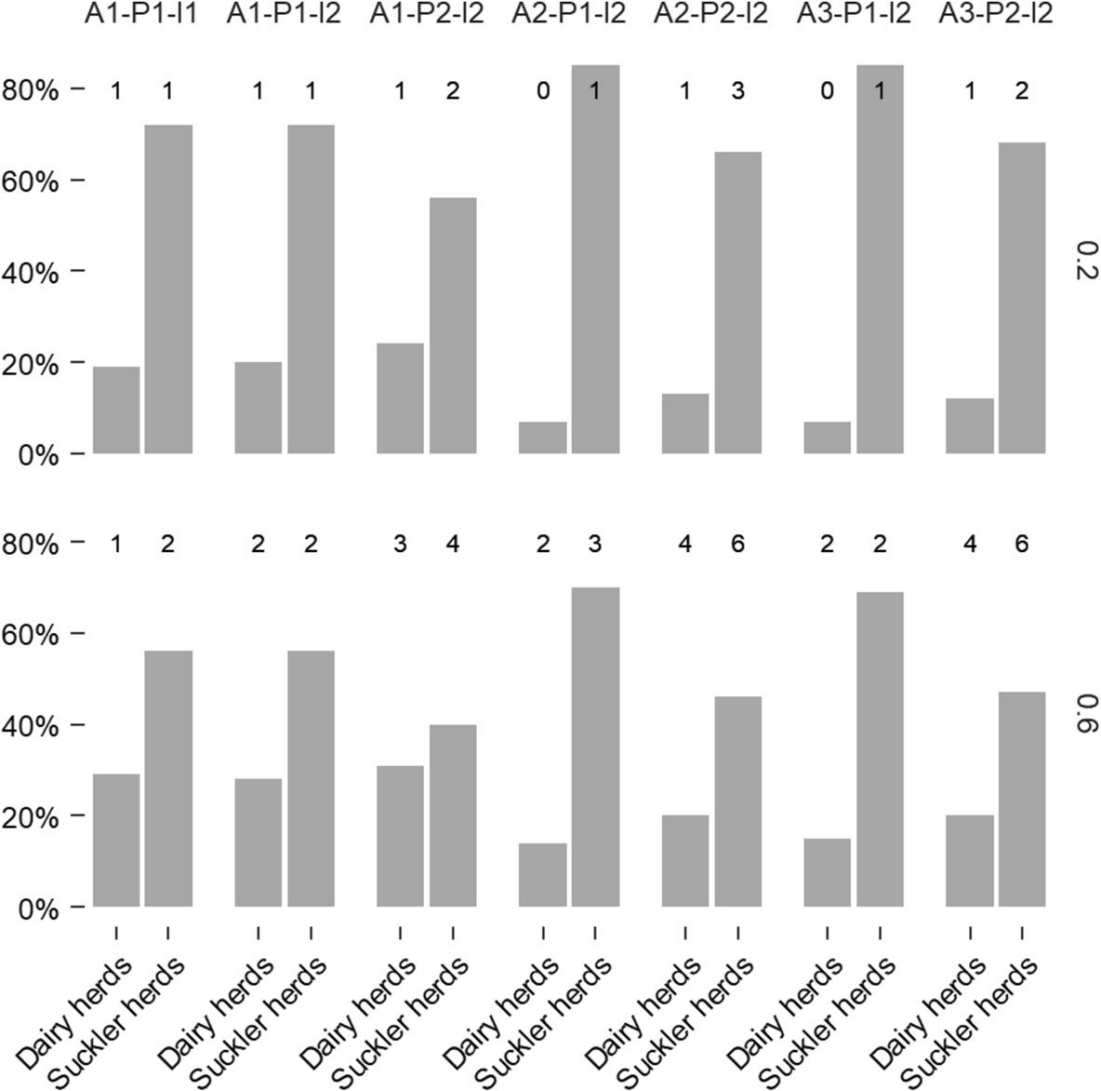
**Average proportion of infected herds detected by the surveillance system, for each production type, surveillance system and epidemiological context (transmission rate).** The numbers above the bars represent the average number of infected herds per simulation for each production type and scenario. Only simulations where infection was detected by the model were considered (72% of all simulations).

The proportion of infected herds under investigation (i.e., detected but not yet confirmed) at the time of infection confirmation was consistently higher in suckler herds than in dairy herds (Figure 2). This difference was more pronounced in the low transmissibility context: on average, 72% of infected suckler herds and 15% of infected dairy herds were recently detected, compared to 55% and 22%, respectively, in the high transmissibility context. Shifting from reporting all abortions (A1) to reporting series of abortions (A2 and A3) amplified this disparity, increasing the proportion of infected suckler herds detected while decreasing the proportion in dairy herds. This effect is likely due to the higher probability of reporting isolated abortions in dairy production (Table 3). Conversely, switching from annual screening of all suckler herds (P1) to one-third of these herds (P2) reduced the gap between production types by decreasing the proportion of detected infected herds in suckler production and increasing it in dairy production (Figure 2).

### Total cost of surveillance and control measures

The number of technical interventions (veterinary visits, samples, and analyses) estimated for each surveillance modality at the national scale, along with their associated costs, are detailed in Additional files 8 and 9.

For the current surveillance system, the total cost was approximately €23 million, regardless of the epidemiological context. This included €22.9 million for annual surveillance and €200,000 for control measures (Table 5). Similarly, for all alternative surveillance-control systems, the total costs were consistent across epidemiological contexts. However, variability in total costs was higher in the high transmissibility context due to greater variability in the number of infected herds at the time of detection (Table 4). Consequently, the epidemiological context is not specified in the following results, as the observations apply to both contexts.

**Table 5:** **Estimated annual surveillance and control costs (million € pre-tax) associated with each scenario.**

Annual cost (million € pre-tax)	Transmission rate	A1-P1-I1	A1-P1-I2	A1-P2-I2	A2-P1-I2	A2-P2-I2	A3-P1-I2	A3-P2-I2
Surveillance	**–**	22.9	19.9	25.0	17.1	22.1	17.1	22.2
Control	0.2	0.2 [0.02–0.7]	0.2 [0.03–0.7]	0.3 [0.04–1.8]	0.2 [0.02–0.5]	0.3 [0.04–1.9]	0.2 [0.02–0.6]	0.3 [0.04–1.7]
	0.6	0.2 [0.04–1.6]	0.2 [0.04–1.7]	0.4 [0.07–4.2]	0.2 [0.03–2.2]	0.7 [0.05–5.8]	0.2 [0.03–2.3]	0.6 [0.06–5.9]
Total	0.2	23.1 [22.9–23.6]	20.1 [19.9–20.6]	25.3 [25.0–26.8]	17.3 [17.1–17.6]	22.4 [22.1–24.0]	17.3 [17.1–17.7]	22.5 [22.2–23.9]
	0.6	23.1 [22.9–24.5]	20.1 [19.9–21.6]	25.4 [25.0–29.2]	17.3 [17.1–19.3]	22.8 [22.2–27.9]	17.3 [17.1–19.4]	22.8 [22.3–28.1]

The costs are presented for each surveillance system and each epidemiological context (transmission rate). The annual cost of surveillance was calculated by adding the estimated cost of each surveillance modality implemented. For control and total costs, the table shows the median, with the first and ninth deciles in square brackets. The total cost was calculated by adding the annual surveillance cost and the median control cost.

The least expensive scenarios, considering both surveillance and control costs, were those excluding screening at introduction (I2), maintaining annual screening of all suckler herds (P1), and requiring mandatory reporting of abortion series (A2 and A3). These scenarios reduced the median total cost by about 26% (€6 million) compared to the current system (A1-P1-I1) (Table 5).

Scenarios with annual surveillance limited to one-third of suckler herds (P2) were associated with higher control costs and were either as costly (A2-P2-I2, A3-P2-I2) or more costly (A1-P2-I2) than the current system. The total costs of these scenarios were consistently about €5 million (25–29%) higher than scenarios with annual screening of all suckler herds (P1), with all other modalities being equal.

Scenarios with mandatory reporting of series of abortions (A2 and A3) had total costs approximately €3 million (12–15%) lower than scenarios requiring mandatory reporting of all abortions (A1), all other modalities being equal. Finally, removing controls at introduction from the current surveillance system (A1-P1-I2) reduced total costs by about 13% (€3 million).

### Identification of the most efficient scenarios

**Figure 3:**
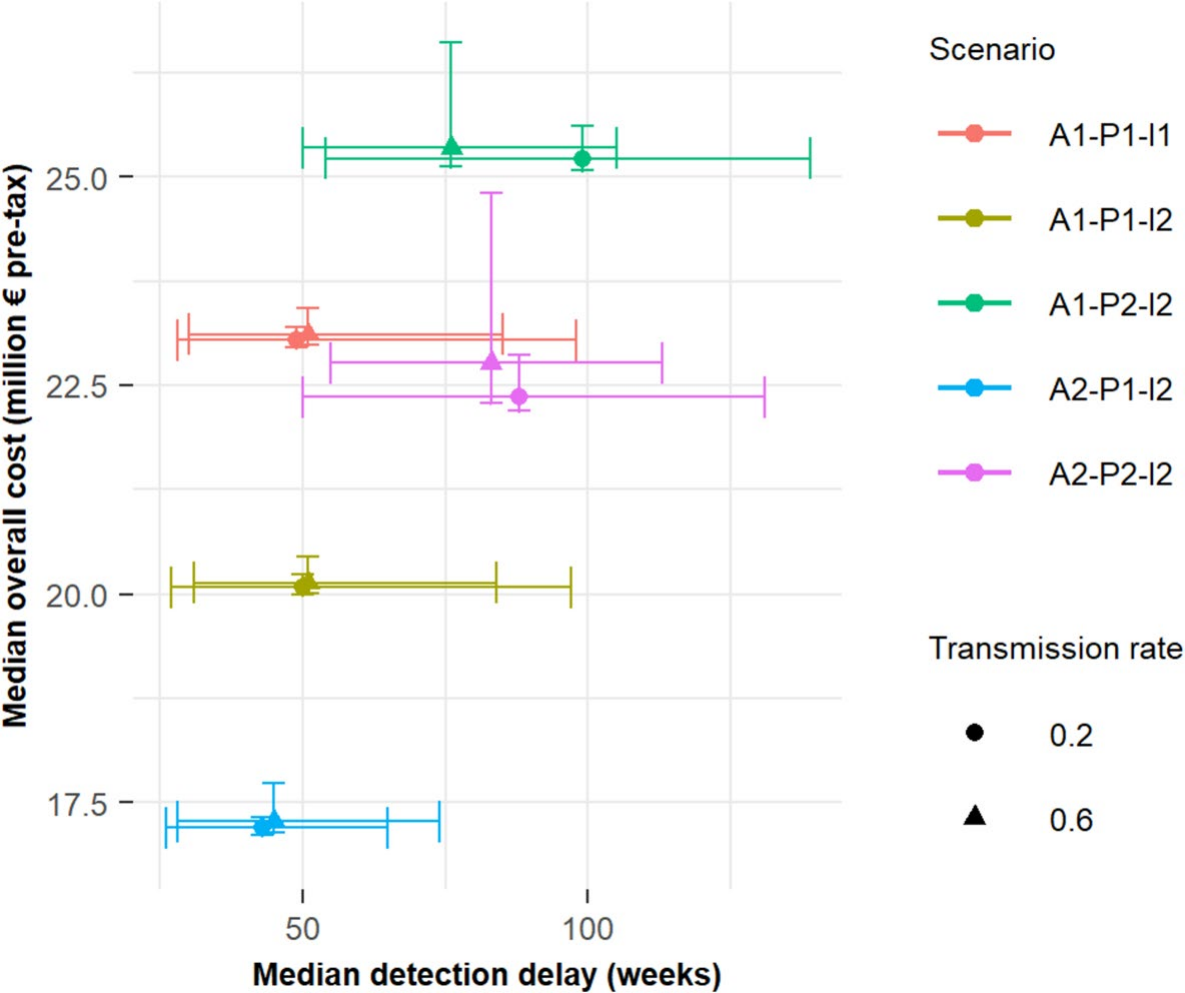
**Relationship between the total cost of surveillance and control (million € pre-tax) and the time to detect infection, for each scenario tested.** Scenarios with the A3-P1-I2 and A3-P2-I2 surveillance systems are not represented because they are superimposed respectively on scenarios A2-P1-I2 and A2-P2-I2 for the corresponding epidemiological context. The horizontal and vertical lines indicate the first and third quartiles.

In both epidemiological contexts, annual screening emerged as the surveillance component with the greatest influence on the cost and effectiveness of the tested surveillance-control systems (Figure 3). Shifting from annual screening of 20% of cattle in all suckler herds (P1) to screening 100% of cattle in one-third of these herds (P2) increased surveillance costs by 25–29% (Table 5) and reduced system performance. This shift resulted in a longer detection delay and a higher number of infected herds at detection, leading to increased control costs.

The removal of controls at introduction improved the efficiency of the surveillance-control system in both epidemiological contexts (Figure 3). Eliminating this component reduced surveillance costs (Table 5) without impacting the system’s performance (Table 4) or control costs (Table 5).

Switching from reporting all abortions (A1) to reporting only series of abortions (A2 and A3) marginally improved the system’s efficiency, reducing total costs by 12–15% without significantly affecting detection delays (Tables 4 and 5, Figure 3). Increasing the probability of reporting series of abortions (A3 versus A2) did not further improve system efficiency under the model’s conditions.

The total cost, detection delay, and thus the efficiency of the surveillance-control system were comparable across both epidemiological contexts for the same surveillance modalities, as indicated by overlapping interquartile ranges (Figure 3). However, the decline in efficiency when shifting from annual screening of all suckler herds (P1) to one-third of these herds (P2), with all other modalities held constant, was more pronounced in the high transmissibility context, due to a significant increase in the median number of infected herds at infection confirmation (Table 4).

Finally, the benefit of switching from reporting all abortions (A1) to reporting series of abortions (A2 and A3) was less pronounced in the high transmissibility context, particularly when combined with the P2 and I2 modalities. In these cases, changing abortion reporting modalities tended to increase both the median number and variability of infected herds at the time of infection confirmation, with all other modalities unchanged (Table 4).

Overall, the A2-P1-I2 scenario (which includes annual herd screening (P1), notification of series of abortions (A2) and no control at introduction (I2)) demonstrated a significantly lower total cost and a shorter detection time compared to other scenarios.

### Sensitivity analysis results

**Figure 4:**
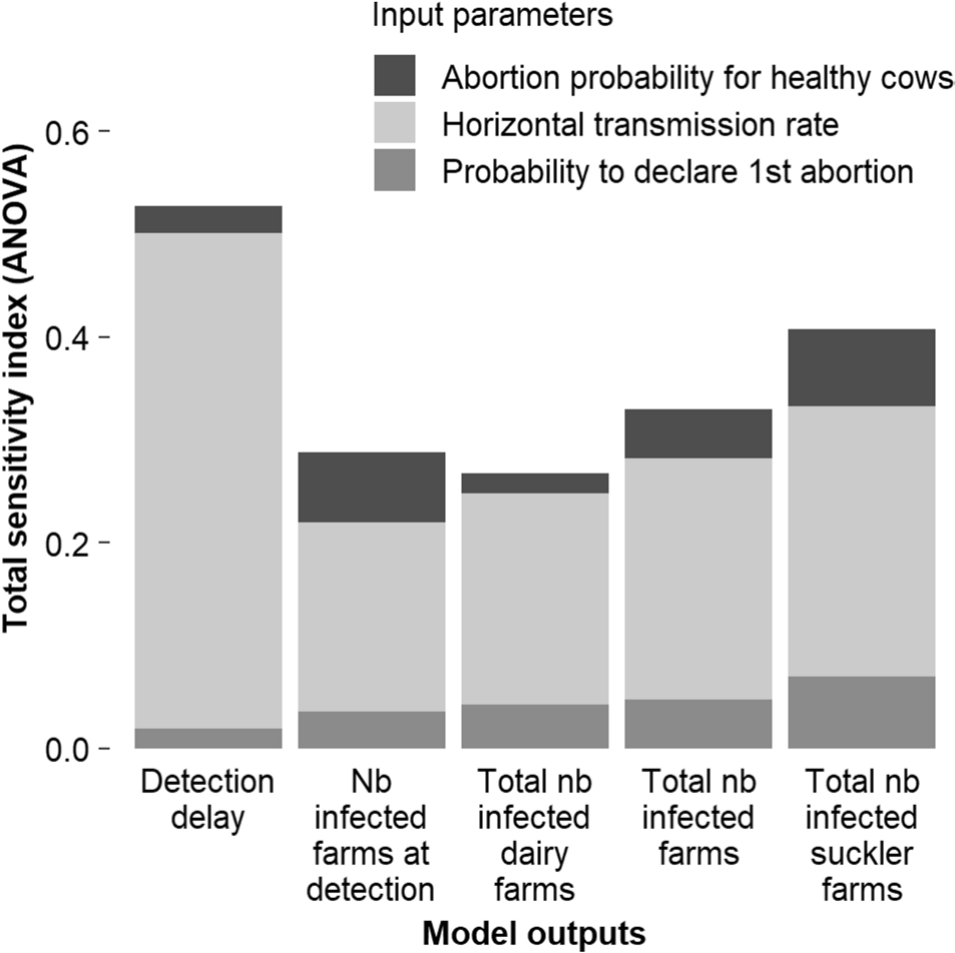
**Contribution (total sensitivity index calculated by an ANOVA) to the variation of target outputs (X axis) of the three model input parameters included in the sensitivity analysis.**

The parameter with the greatest influence on the model outputs was the horizontal transmission rate, which accounted for 48% of the variability in detection delay and 26% of the variability in the number of infected herds at the time of confirmation of infection (Figure 4). The probability of abortion in healthy females and the probability of reporting the first abortion also influenced the outputs, contributing 4–15% of the output variability when combined. In contrast, the probability of abortion in infected females had no significant effect on the model outputs. The probability of selling an animal to a farm of the same production type had no influence on the outputs.

It is noteworthy that 50–75% of the variability in the output parameter values remained unexplained. The negligible interaction between the tested parameters does not account for this residual variability. Instead, it is likely attributable to variability in input parameters not included in the sensitivity analysis (e.g., age at first insemination, the duration of gestation, postpartum periods, or high-shedding periods) or to intrinsic variability in the model itself. Examples of this intrinsic variability include the physiological states of females that are purchased or lent, the distribution of age and physiological states of animals when an infected animal is introduced into a farm, and the selection of farms introducing an infected animal.

## Discussion

This study aimed to evaluate the efficiency of current and alternative surveillance-control systems for bovine brucellosis in France, in the context of its disease-free status, following the introduction of an infected animal through purchase. A mechanistic, stochastic, agent-based, discrete-time simulation model was developed to test seven surveillance systems under two epidemiological contexts (low and high transmissibility). All systems complied with European regulations [6] and were informed by surveillance strategies implemented in European countries [8].

The model assessed the ability of the current surveillance system to detect an outbreak and explored improvements to its efficiency. Efficiency was compared using two performance indicators—detection delay and the number of infected farms at the first confirmation of infection—and by simulating total surveillance and control costs. The current system detected infection within a median delay of 49 weeks, with one to three infected farms at confirmation in 90% of simulations. No tested system was more effective in terms of detection, as the median number of infected farms at confirmation ranged from one to three across all systems and contexts. However, alternative systems showed potential for cost reduction. In particular, the A2-P1-I2 scenario, which investigated series of abortions (A2), annual herd screening (P1) and no controls at introduction (I2), presents the best compromise between cost and effectiveness.

### Detection probability and delay

Our results showed that infected suckler herds were proportionally more likely to be detected than dairy herds, primarily through annual herd screening. This is likely due to the surveillance measures modeled, where the annual selection of animals for testing in suckler herds prioritized those at higher risk (i.e. introduced recently without test at purchase), as recommended by the Ministry of Agriculture [40]. The diagnosis of brucellosis on a large scale is based on series of serological tests, whose variations in accuracy may occur depending on the test itself and the epidemiology of brucellosis [40, 41]. Although the choice of Se and Sp values [40] may influence the detection probability and therefore the detection delay, we believe that it had no marked influence on our results and on the comparison of scenarios given that our model reproduced the series of screening and confirmatory tests and used the same diagnostic performances for all scenarios.

We observed a decrease in surveillance efficiency when screening only one-third of suckler herds annually (P2) compared to screening all herds (P1). In this study, P2 was modeled as a random selection of all suckler herds for screening once every three years, a practice already implemented in some European countries [8]. As suggested in previous work [19], performance of partial screening could be improved by selecting all at-risk herds annually and randomly selecting additional herds to reach one-third of the total suckler herds. In France, at-risk herds represent 6–10% of the population (BDNI database) and include herds with characteristics such as: (1) production or practices increasing zoonotic risk (e.g., raw milk and unpasteurized cheese production); (2) high turnover rates increasing brucellosis introduction risk; (3) links to previous outbreaks or wildlife reservoirs (e.g., *Capra ibex* in the Alps); or 4) identified deficiencies in health risk management by veterinary services [7]. This approach would ensure annual screening of at-risk herds, with the remaining herds (20–24%) being screened at least once every four to five years. While our study did not explicitly test this risk-based surveillance strategy, further research incorporating additional data is needed to evaluate its feasibility and relevance.

Virtually no infected herds were detected through controls at introduction, consistent with earlier findings [19]. This is likely due to the low probability of testing at introduction (0.06 for non-at-risk farms versus 1 for at-risk farms, which constitute only 6% of the sample). A potential improvement could involve reinstating introduction screening as part of control measures following the detection of an initial case. This could facilitate identification of secondary outbreaks, particularly in high transmission or endemic contexts, improving disease containment. However, such measures fall outside the scope of this study.

Annual risk-based screening would likely result in higher surveillance costs for producers managing at-risk herds. To address this disparity, pooling costs at the departmental level through health support organizations could ensure fairness and encourage participation. Although not tested in this study, this approach warrants further exploration in future research.

### Improved clinical surveillance

Our study demonstrated that focusing on the reporting of abortion series rather than all abortions could enhance the overall efficiency of the surveillance-control system, primarily by reducing total costs without affecting detection delay. This finding aligns with previous modeling studies [19] and reflects practices in other countries aimed at improving farmers’ reporting rates [8].

The simulated increase in the probability of reporting abortion series (A3 versus A2) did not influence system efficiency under the assumptions of our model. This result may stem from the low occurrence of abortion series and the limited contribution of clinical surveillance to overall system performance, which is affected by the significant under-reporting of abortions [13]. Investigating the occurrence of abortion series in brucellosis-free areas and in regions where the disease is spreading, under varying epidemiological contexts, could refine the modeling and better evaluate the scenarios. Moreover, examining the factors influencing farmers’ decisions to report single abortions versus series of abortions [31] would further improve understanding and simulation accuracy.

The predicted cost savings from shifting from reporting all abortions (A1) to reporting series of abortions (A2 or A3) could be reallocated to enhance the effectiveness of clinical surveillance. For instance, these resources could support increased farmer awareness about the importance of reporting abortion series and conducting diagnostic tests for brucellosis and other abortion-related diseases, whether regulated or not.

However, specific production systems, such as those involving unpasteurized milk, may still require investigation of all detected abortions to identify pathogens other than *Brucella* spp. If reporting single abortions becomes non-mandatory, it is crucial for the government to continue funding veterinary visits, sampling, and analyses for isolated abortions. This would encourage farmers to report any suspicious abortion cases, thereby maintaining vigilance for other potential threats.

### Specificities of our model

This study evaluated the effectiveness of each tested surveillance system in relation to its total cost. Costs associated with surveillance systems are often neglected in modeling studies [20, 22], primarily due to the lack of economic data. By incorporating these costs, this study provides additional insights into potential improvements for bovine brucellosis surveillance, considering both surveillance and control expenses for each scenario. A prior study assessed the cost-effectiveness of the French surveillance system using a scenario tree approach [19]. While the scenario tree method effectively evaluates a surveillance system's ability to demonstrate disease absence [42], it cannot assess the detection delay following potential disease reintroduction. To address this limitation, we developed an epizootic simulation model to enhance accuracy and provide complementary insights. By incorporating animal movements and modelling disease spread dynamics, this model allowed us to estimate control costs in addition to surveillance costs. This broader perspective equips decision-makers with detailed information on the performance of surveillance-control systems, potential improvements, and the cost or zoonotic risk implications of proposed changes.

Our model showed that with the current surveillance system, the median detection delay for a bovine brucellosis outbreak was 49 weeks, with one to three farms infected at first confirmation in 90% of simulations. Alternative surveillance systems yielded similar results, with one to 27 farms infected in 90% of cases under the worst scenario (high transmissibility, partial annual screening (P2), declaration of series of abortions (A2 or A3), and no screening at introduction (I2)). Out of approximately 155 000 farms in France in 2020–2021 [1], this represents a proportion of infected farms below 0.02%. The zoonotic risk posed by an infected herd primarily occurs during the high-shedding period in the two months following calving or abortion by infected females. These findings suggest that under the model's assumptions and tested epidemiological contexts, the risk of human contamination remains relatively low for all surveillance systems.

The model explicitly accounted for movements of brucellosis-infected bovines, introducing new farms into the simulation. This approach avoided the need to map all exchanges between French farms or to model non-infected farms while introducing stochasticity in transmission routes compared to a fixed network of farm movements. Studies indicate no consistent pattern in the characteristics of farms linked by animal exchanges [43, 44], apart from a higher likelihood of movements occurring between farms of the same production type [45], which our model included. While mechanistic models of trade movements, such as for veal calves, show promise, they currently provide robust predictions only for large trading units over extended periods [46], suggesting potential for future integration in medium- or long-term studies.

We did not include potential stays of animals in assembly centers or markets, as data on these intermediaries are incomplete and challenging to use for modeling. Studies indicate that half of animal movements involve intermediate traders [47]. However, animals typically stay briefly in these centers and are rarely in the postpartum period, which is the high-shedding phase for infected animals. Therefore, the risk of bovine brucellosis transmission in these locations is considered relatively low.

### Limitations of the epidemiological component of the model

We simulated two epidemiological contexts (low and high transmissibility rates) to account for variability in brucellosis transmissibility among *Brucella* spp. strains [27, 48], as well as the capacity of bovine brucellosis to cause either sporadic or serial abortions [3, 11]. Given that France has been officially free of bovine brucellosis since 2005, with only two outbreaks in 2012 and one in 2021—each contained within a single farm—there is no national data to validate the epidemiological dynamics of our model. However, the model outcomes align with data from European outbreaks in Ireland and Belgium [14, 16, 17]. Sensitivity analysis showed that the outcomes of the surveillance-control systems were most influenced by the horizontal transmission rate, highlighting the importance of refining this parameter as more data becomes available.

In the model, horizontal transmission rates for high-shedding females were set at 0.2 and 0.6 per week, reflecting strain diversity. High-shedding was assumed to occur for approximately nine weeks post-calving, followed by low-shedding for about 45 weeks until the next calving, depending on breed. The low-shedding transmission rate was set at one-tenth of the high-shedding rate, resulting in average weekly transmission rates of 0.05 and 0.15. These values are consistent with existing brucellosis models [20, 21, 29, 30], despite the lack of specific data to validate them. The model did not include a latency period (from infection to infectiousness) due to limited literature evidence. Latency is considered infrequent, with up to 10% of calves born to infected females developing latent infections [49, 50].

Transmission through neighborhood contacts, such as during grazing, transhumance, or indirect exposure via contaminated equipment, vehicles, or personnel, was not included in the model. This exclusion may underestimate the spread of brucellosis between herds. The lack of data on this transmission mode in the context of French agriculture makes parameter estimation difficult.

Males were also excluded from the model, as their contribution to brucellosis spread is assumed to be minimal for three reasons: breeding bulls and their semen are strictly controlled in France, resulting in negligible transmission risk; venereal transmission of brucellosis is considered very rare [28]; and male calves are typically either slaughtered before sexual maturity or become breeding bulls, with both pathways involving minimal bacterial shedding.

### Cost estimates: limitation and interpretation

The total costs of each surveillance-control system were based on available data regarding the costs of analyses, technical interventions [12], and government compensation for infected farms [36, 37]. However, these estimates are likely underestimated as they do not account for the human and material resources required for operating the surveillance system and implementing control measures. These include infrastructure, maintenance, training, network coordination, and epidemiological investigations, managed by the organizations providing health support to livestock farmers (known as "GDS") and veterinary services. These costs have never been quantified, though administrative management costs are likely similar across all scenarios.

For control costs, additional compensation or direct and indirect costs for farmers not covered by compensation were excluded due to their high variability and lack of data. Furthermore, our model showed no significant differences in the number of infected herds at the time of confirmation between surveillance-control systems in the low transmissibility context and only small differences in the high transmissibility context (with a median of up to two more infected herds for comparable systems). Given that control costs constitute a minor portion of total costs, these exclusions are unlikely to affect the comparative efficiency of the surveillance-control systems.

In France, annual screening visits and controls at introduction often test for multiple diseases, both regulated and unregulated, alongside bovine brucellosis. Shared sampling reduces the specific costs attributed to brucellosis surveillance. One study estimated that 30% of veterinary costs associated with the current brucellosis surveillance system (visits and blood samples) are shared with other regulated diseases such as bovine tuberculosis, infectious bovine rhinotracheitis (IBR), and enzootic bovine leukosis [12, 19]. Consequently, potential savings in the brucellosis surveillance system from reduced veterinary costs would be lower than estimated here. For instance, implementing annual screening of only one-third of suckler farms (instead of all farms in the current system) would not eliminate the costs of visits and blood sampling for the remaining two-thirds. These farms would still incur costs for IBR testing (annual blood screening of all suckler farms) or bovine tuberculosis testing (annual allergen testing for at-risk farms). Only the costs related to enzootic bovine leukosis surveillance, which involves annual serological analysis in 20% of suckler farms, could remain coupled with brucellosis screening under such a scenario.

Our results indicate that, under the assumptions of the model and across the tested epidemiological contexts, all surveillance-control systems maintained a relatively low number of infected herds. Even in the worst-case scenario—characterized by the highest horizontal transmission rate and the lightest surveillance system—less than 0.02% of herds were infected after a four-year period in 90% of simulations. No significant differences in effectiveness were observed among the seven tested surveillance-control systems in either low or high transmissibility contexts. However, the alternative surveillance modalities had specific effects on the overall efficiency of the surveillance-control system. Efficiency improved when controls at introduction were stopped, decreased when only one-third of suckler herds (randomly selected) were screened annually instead of all, and slightly improved when reporting series of abortions (two or more in one month) rather than all abortions.

## Supplementary Information

**Additional file 1: Selection process of representative farms from the French national cattle identification database (BDNI).** This selection was used to define the demographic characteristics of the simulated farms, based on real data recorded for each week over the period July 2013-June 2017 (total number of animals, demographics by age state, number and age state of the animals that die, are slaughtered, sold/lent or purchased/hired).

**Additional file 2: Description of a “state machine” in the simulation model **[24]**.**

**Additional file 3:** **Graphic representation of the main processes of the model at individual level.** Solid arrows represent changes of state; dashed arrows represent the influence some processes have on other processes. In the Serology frame, the green solid arrows refer to annual screening and post-abortion tests, and the red solid arrows refer to the tests realized on purchase. The blue frames are the state machines that constitute the demographic compartment of the model, the red frames constitute the epidemiological compartment and the green frames constitute the surveillance compartment. Animals are either introduced during the simulation, in which case they can be tested on purchase with a given probability, or present in the herd at the time of its introduction in the simulation, in which case they are in the state “Untested” and can be tested in the annual screening with a given probability. Vertical and horizontal transmission of disease to other animals are not represented.

**Additional file 4: Evolution of the real (dotted lines) and simulated (continuous lines) herd sizes according to time (in weeks) for twenty herds of each production type (dairy and suckler), during simulations without any infection.**

**Additional file 5: Ratios of the difference between simulated and actual herd sizes to actual herd sizes at the end of the simulation period for the 610 sample farms in our final sample.** The graph shows the value of this ratio for suckler (left) and dairy (right) farms. The percentages shown above each box plot correspond to the proportions of farms selected from the simulated farms, for each type of production.

**Additional file 6: Veterinary fees used for the evaluation of surveillance costs, according to the surveillance component.**

**Additional file 7: Unit costs of laboratory analyses, used to assess monitoring costs (median and interquartile ranges).**

**Additional file 8: Estimated annual number of technical interventions and analyses carried out at the national level, for each modality of the three monitoring systems.**

**Additional file 9: Estimated annual costs (€ pre-tax) of technical interventions and analyses carried out at national level, for each modality of the three monitoring schemes.**

## Data Availability

The data that support the findings of this study are available from French Minsitry of Agriculture but restrictions apply to the availability of these data and so they are not publicly available. The description of the model is available online [26].
