# Supplementary material for: Assessing the efficiency of the bovine brucellosis surveillance-control system in a disease-free context through agent-based modelling
Source: Vet Res. 2025 Jun 17;56:120. doi: 10.1186/s13567-025-01549-1 (PMC12172338; doi:10.1186/s13567-025-01549-1)

**Additional file 1. Selection process of representative farms from the French national cattle identification database (BDNI).**

This selection was used to define the demographic characteristics of the simulated farms, based on real data recorded for each week over the period July 2013-June 2017 (total number of animals, demographics by age state, number and age state of the animals that die, are slaughtered, sold/lent or purchased/hired).


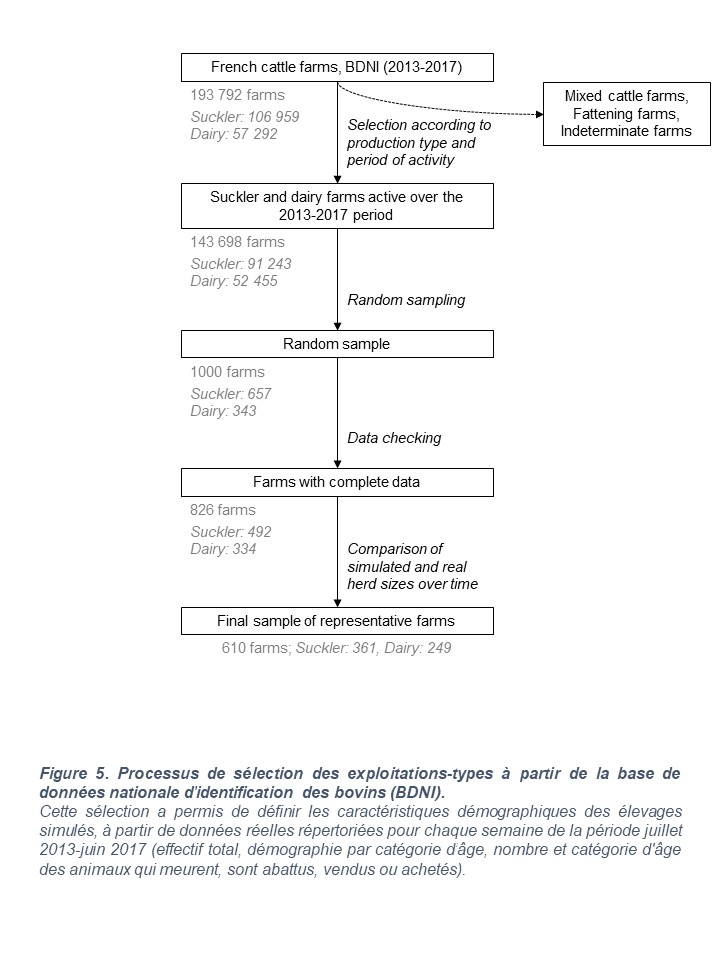

Supplement: Supplementary file 1 — Additional file 1: Selection process of representative farms from the French national cattle identification database (BDNI). This selection was used to define the demographic characteristics of the simulated farms, based on real data recorded for each week over the period July 2013-June 2017 (total number of animals, demographics by age state, number and age state of the animals that die, are slaughtered, sold/lent or purchased/hired). [file 13567_2025_1549_MOESM1_ESM.docx]
