# Supplementary material for: Assessing the efficiency of the bovine brucellosis surveillance-control system in a disease-free context through agent-based modelling
Source: Vet Res. 2025 Jun 17;56:120. doi: 10.1186/s13567-025-01549-1 (PMC12172338; doi:10.1186/s13567-025-01549-1)
Supplement: Supplementary file 2 — Additional file 2: Description of a “state machine” in the simulation model [24]. [file 13567_2025_1549_MOESM2_ESM.docx]

**Additional file 2. Description of a “state machine” in the simulation model [24].**

In the EMULSION model, processes called "state machines" (a computer science formalism equivalent to the flow diagrams of conventional epidemiological models) manage the dynamics of individual demographic, epidemiological and surveillance-control characteristics, which are taken into account in the first three components of the model. Each state machine is designed with specific states, transitions, conditions, durations and values of rates or probabilities, depending on the modelling assumptions.

The components of the model (parameters, hypotheses, processes, data) were expressed in the form of a structured text file (in YAML language) and processed by the generic simulation engine EMULSION (1.1 version). This document is available on request.


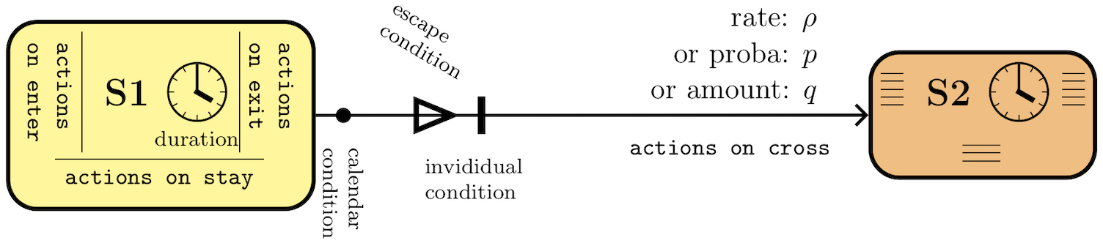
Example :

In our model, a state machine managed the level of shedding of *Brucella* bacteria by infected cattle. This machine had three states: non-shedder, low shedder, and high shedder. All healthy cattle had the non-shedder status. If they were infected, they systematically became low shedders at the time of infection and high shedders at the time of calving or abortion. They then remained high shedders for nine weeks, after which they automatically became low shedders again.
