# Supplementary material for: Assessing the efficiency of the bovine brucellosis surveillance-control system in a disease-free context through agent-based modelling
Source: Vet Res. 2025 Jun 17;56:120. doi: 10.1186/s13567-025-01549-1 (PMC12172338; doi:10.1186/s13567-025-01549-1)
Supplement: Supplementary file 3 — Additional file 3: Graphic representation of the main processes of the model at individual level. Solid arrows represent changes of state; dashed arrows represent the influence some processes have on other processes. In the Serology frame, the green solid arrows refer to annual screening and post-abortion tests, and the red solid arrows refer to the tests realized on purchase. The blue frames are the state machines that constitute the demographic compartment of the model, the red frames constitute the epidemiological compartment and the green frames constitute the surveillance compartment. Animals are either introduced during the simulation, in which case they can be tested on purchase with a given probability, or present in the herd at the time of its introduction in the simulation, in which case they are in the state “Untested” and can be tested in the annual screening with a given probability. Vertical and horizontal transmission of disease to other animals are not represented. [file 13567_2025_1549_MOESM3_ESM.docx]

**Additional file 3. Graphic representation of the main processes of the model at individual level.** Solid arrows represent changes of state; dashed arrows represent the influence some processes have on other processes. In the Serology frame, the green solid arrows refer to annual screening and post-abortion tests, and the red solid arrows refer to the tests realized on purchase. The blue frames are the state machines that constitute the demographic compartment of the model, the red frames constitute the epidemiological compartment and the green frames constitute the surveillance compartment. Animals are either introduced during the simulation, in which case they can be tested on purchase with a given probability, or present in the herd at the time of its introduction in the simulation, in which case they are in the state “Untested” and can be tested in the annual screening with a given probability. Vertical and horizontal transmission of disease to other animals are not represented.

**
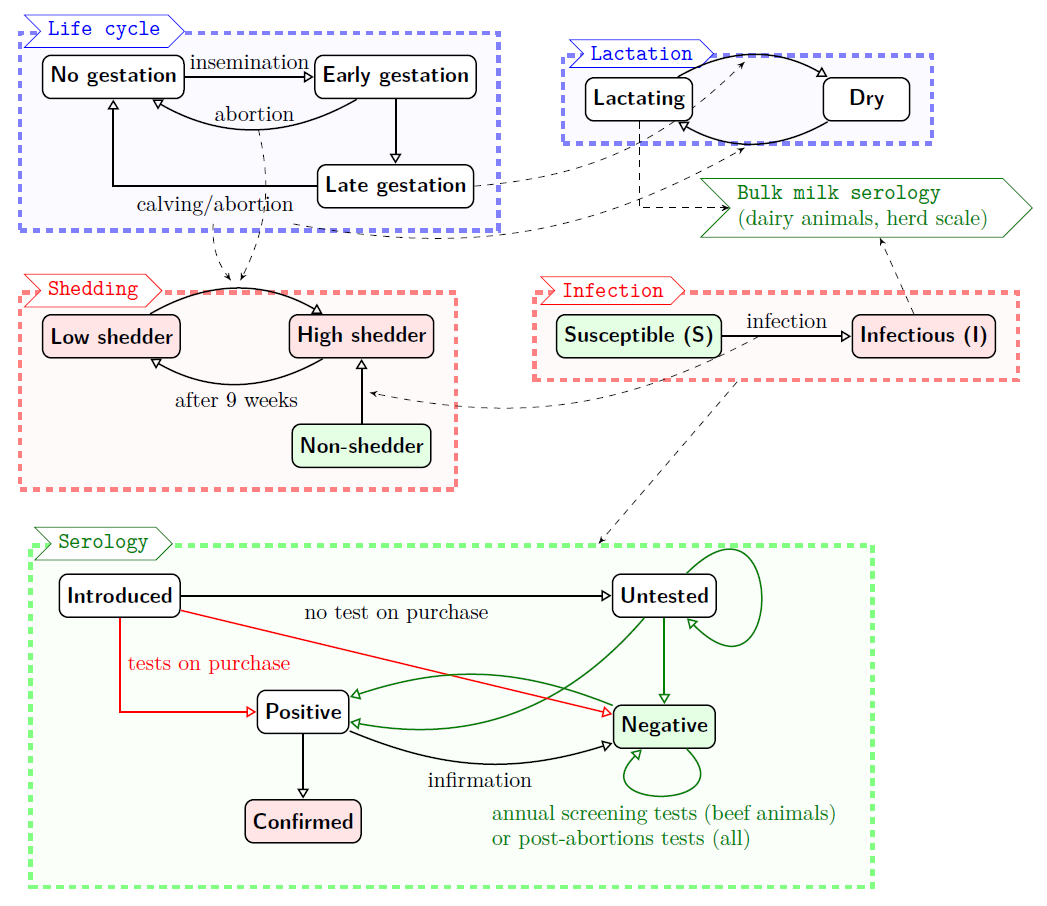
**
