# Supplementary material for: Assessing the efficiency of the bovine brucellosis surveillance-control system in a disease-free context through agent-based modelling
Source: Vet Res. 2025 Jun 17;56:120. doi: 10.1186/s13567-025-01549-1 (PMC12172338; doi:10.1186/s13567-025-01549-1)

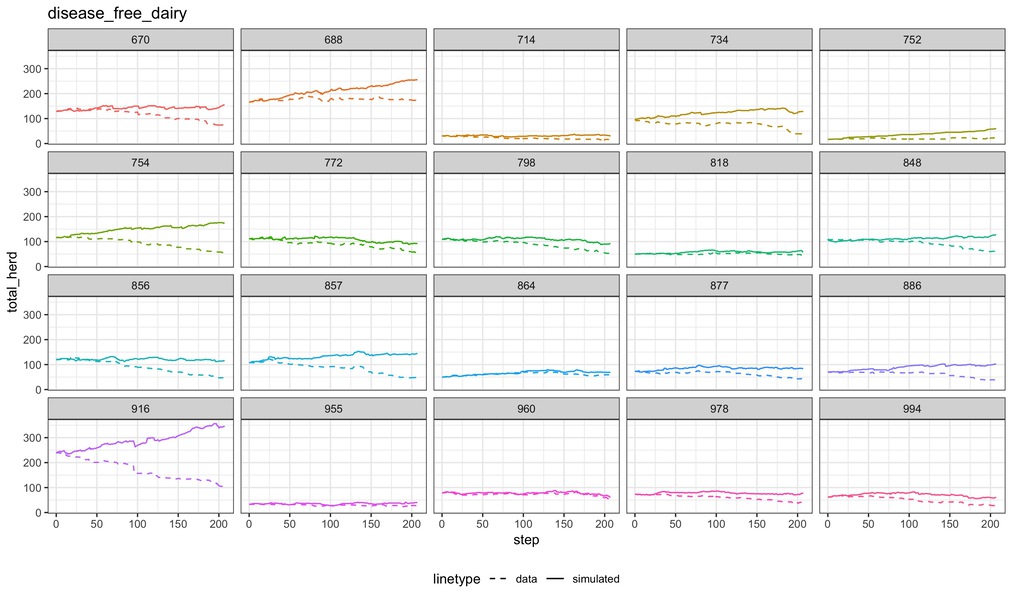
Additional file 4. Evolution of the real (dotted lines) and simulated (continuous lines) herd sizes according to time (in weeks) for twenty herds of each production type (dairy and suckler), during simulations without any infection.


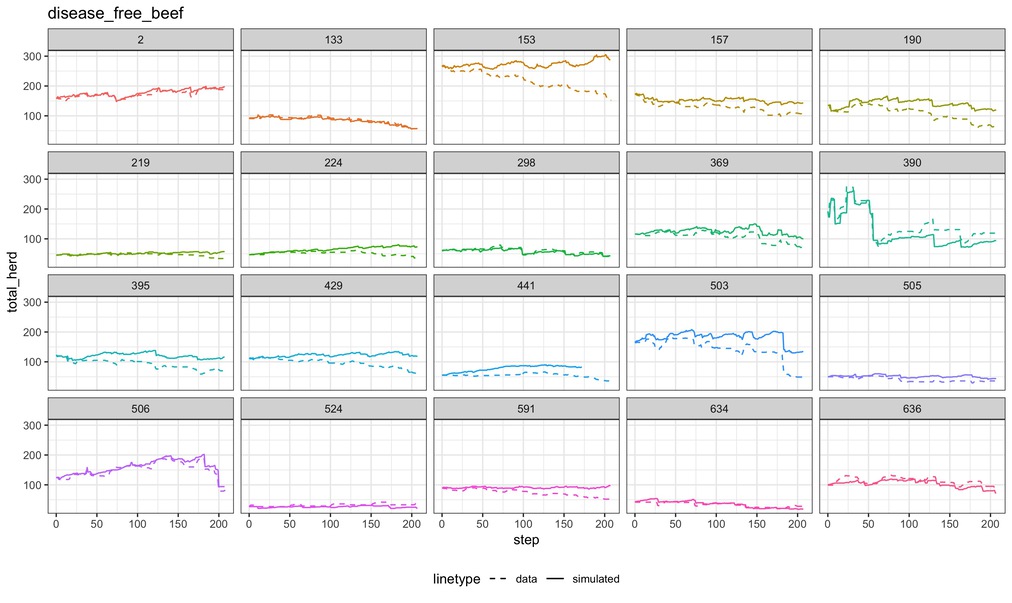

Supplement: Supplementary file 4 — Additional file 4: Evolution of the real (dotted lines) and simulated (continuous lines) herd sizes according to time (in weeks) for twenty herds of each production type (dairy and suckler), during simulations without any infection. [file 13567_2025_1549_MOESM4_ESM.docx]
