# Supplementary material for: Assessing the efficiency of the bovine brucellosis surveillance-control system in a disease-free context through agent-based modelling
Source: Vet Res. 2025 Jun 17;56:120. doi: 10.1186/s13567-025-01549-1 (PMC12172338; doi:10.1186/s13567-025-01549-1)

**Additional file 5. Ratios of the difference between simulated and actual herd sizes to actual herd sizes at the end of the simulation period for the 610 sample farms in our final sample.** The graph shows the value of this ratio for suckler (left) and dairy (right) farms. The percentages shown above each box plot correspond to the proportions of farms selected from the simulated farms, for each type of production.


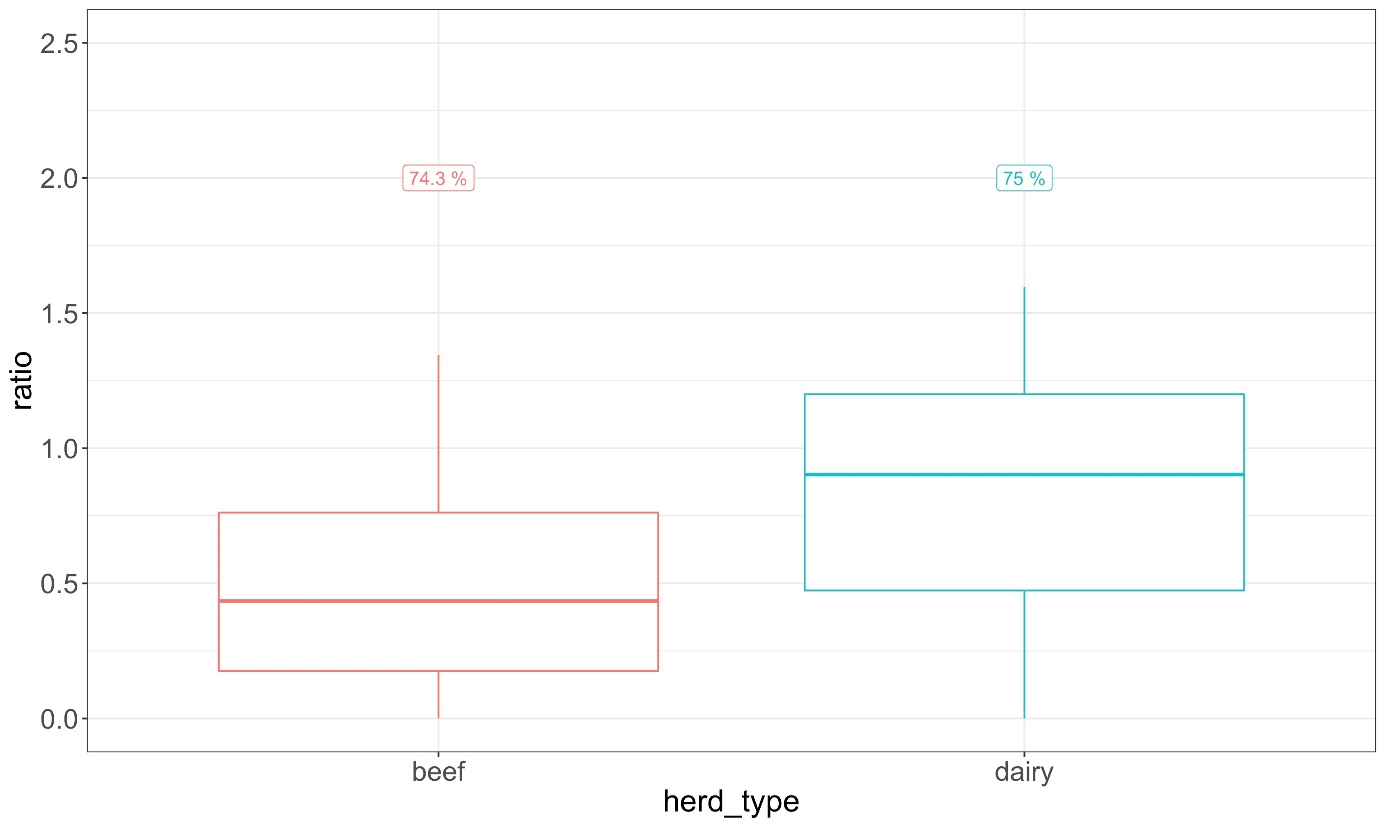

Supplement: Supplementary file 5 — Additional file 5: Ratios of the difference between simulated and actual herd sizes to actual herd sizes at the end of the simulation period for the 610 sample farms in our final sample. The graph shows the value of this ratio for suckler (left) and dairy (right) farms. The percentages shown above each box plot correspond to the proportions of farms selected from the simulated farms, for each type of production. [file 13567_2025_1549_MOESM5_ESM.docx]
