# Supplementary material for: Assessing the efficiency of the bovine brucellosis surveillance-control system in a disease-free context through agent-based modelling
Source: Vet Res. 2025 Jun 17;56:120. doi: 10.1186/s13567-025-01549-1 (PMC12172338; doi:10.1186/s13567-025-01549-1)
Supplement: Supplementary file 6 — Additional file 6: Veterinary fees used for the evaluation of surveillance costs, according to the surveillance component. [file 13567_2025_1549_MOESM6_ESM.docx]

**Additional file 6. Veterinary fees used for the evaluation of surveillance costs, according to the surveillance component.** Values (in € pre-tax) are based on [12], corrected to take into account the increase in the monetary unit of the AMV.

| **Intervention** | **Abortion reporting** | **Annual screening** | **Testing at introduction** |
| --- | --- | --- | --- |
| Veterinarian’s visit | 28.36 | 24.33 | 28.36 |
| Sampling of genital organs, placenta, foetus for bacteriology (per sampled animal) | 7.09 | - | - |
| Sample for serological testing (per sampled animal) | 2.84 | 2.34 | 2.42 |
| Travel allowances (per kilometre) | 0.32 | - | - |
| Compensation for travel time (per kilometre) | 0.95 | - | - |
| Lump-sum compensation of expenses and travel time | - | 8.83 | 13.70 |
