# Supplementary material for: Assessing the efficiency of the bovine brucellosis surveillance-control system in a disease-free context through agent-based modelling
Source: Vet Res. 2025 Jun 17;56:120. doi: 10.1186/s13567-025-01549-1 (PMC12172338; doi:10.1186/s13567-025-01549-1)
Supplement: Supplementary file 7 — Additional file 7: Unit costs of laboratory analyses, used to assess monitoring costs (median and interquartile ranges). [file 13567_2025_1549_MOESM7_ESM.docx]

**Additional file 7. Unit costs of laboratory analyses, used to assess monitoring costs (median and interquartile ranges).** Values are based on [12].

| **Method of analysis** | **Median unit cost (€ pre-tax)**  **[interquartile range]** |
| --- | --- |
| Bacteriology | 75.06 [47.98 - 89.25] |
| Rose Bengal test | 2.02 [1.77 - 2.60] |
| Complement fixation test | 6.52 [5.59 - 8.71] |
| ELISA on individual serum | 7.00 [5.75 - 7.47] |
| ELISA on tank milk | 3.25 [2.89 - 5.12] |
| ELISA on mixed sera | 8.91 [6.17 - 11.5] |
