# Supplementary material for: Assessing the efficiency of the bovine brucellosis surveillance-control system in a disease-free context through agent-based modelling
Source: Vet Res. 2025 Jun 17;56:120. doi: 10.1186/s13567-025-01549-1 (PMC12172338; doi:10.1186/s13567-025-01549-1)
Supplement: Supplementary file 8 — Additional file 8: Estimated annual number of technical interventions and analyses carried out at the national level, for each modality of the three monitoring systems. [file 13567_2025_1549_MOESM8_ESM.docx]

**Additional file 8. Estimated annual number of technical interventions and analyses carried out at the national level, for each modality of the three monitoring systems.**

| **Intervention / Analysis** | **A1** | **A2** | **A3** | **P1 (suckler)** | **P1,P2 (dairy)** | **P2 (suckler)** | **I1** | **I2** |
| --- | --- | --- | --- | --- | --- | --- | --- | --- |
| Veterinary visits | 60 578 | 10 601 | 11 605 | 125 139 | - | 41 713 | 48 064 | 0 |
| Samples | 58 213^(1)^ | 20 375^(1)^ | 22 305^(1)^ | 1 453 004^(1)^ | - | 2 421 673^(1)^ | 138 399^(1)^ | 0 |
|  | 11 305c | 3 957^(2)^ | 4 332^(2)^ |  |  |  |  |  |
| ELISA on tank milk | - | - | - | - | 62 949 | - | - | - |
| Individual serum ELISA | 21 685 | 5 931 | 6 493 | 21 266 | - | 35 070 | 33 146 | 0 |
| ELISA on mixed sera | - |  |  | 793 412 | - | 1 308 408 | 10 975 | 0 |
| Rose Bengal test | 55 836 | 15 271 | 16 718 | 653 812 | - | 1 078 195 | 141 855 | 0 |
| Complement fixation test | 6 957 | 2 435 | 2 666 | 1 474 | - | 2 457 | 784 | 0 |
| Bacteriology | 2 391 | 837 | 916 | - | - | - | - | - |

*^(1)^ Blood samples; ^(2)^ Samples of genital organs (female), placenta or foetus*

*ELISA: enzyme-linked immunosorbent assay*
