# Supplementary material for: Assessing the efficiency of the bovine brucellosis surveillance-control system in a disease-free context through agent-based modelling
Source: Vet Res. 2025 Jun 17;56:120. doi: 10.1186/s13567-025-01549-1 (PMC12172338; doi:10.1186/s13567-025-01549-1)
Supplement: Supplementary file 9 — Additional file 9: Estimated annual costs (€ pre-tax) of technical interventions and analyses carried out at national level, for each modality of the three monitoring schemes. [file 13567_2025_1549_MOESM9_ESM.docx]

**Additional file 9. Estimated annual costs (€ pre-tax) of technical interventions and analyses carried out at national level, for each modality of the three monitoring schemes.**

| **Intervention / Analysis** | **A1** | **A2** | **A3** | **P1 (suckler)** | **P1,P2 (dairy)** | **P2 (suckler)** | **I1** | **I2** |
| --- | --- | --- | --- | --- | --- | --- | --- | --- |
| Veterinary visits | 2 867 763 | 501 858 | 549 400 | 4 148 547 | - | 1 382 849 | 2 021 514 | 0 |
| Samples | 165 092^(1)^ | 57 782^(1)^ | 63 256^(1)^ | 3 406 660^(1)^ | - | 5 677 766^(1)^ | 334 404^(1)^ | 0 |
|  | 80 152^(2)^ | 28 053^(2)^ | 30 711^(2)^ |  |  |  |  |  |
| ELISA on tank milk | - | - | - | - | 204 270 | - | - | - |
| Individual serum ELISA | 151 795 | 41 517 | 45 449 | 148 862 | - | 245 487 | 232 022 | 0 |
| ELISA on mixed sera |  |  |  | 7 069 301 | - | 11 657 919 | 97 787 | 0 |
| Rose Bengal test | 112 789 | 30 848 | 33 770 | 1 320 700 | - | 2 177 955 | 286 547 | 0 |
| Complement fixation test | 45 360 | 15 876 | 17 380 | 9 610 | - | 16 017 | 5 112 | 0 |
| Bacteriology | 179 468 | 62 814 | 68 764 | - | - | - | - | - |
| **TOTAL** | **3 602 419** | **738 749** | **808 730** | **16 103 680** | **204 270** | **21 157 993** | **2 977 386** | **0** |

*^(1)^ Blood samples; ^(2)^ Samples of genital organs (female), placenta or foetus*

*ELISA: enzyme-linked immunosorbent assay*
